# Aromatase Deficiency, a Rare Syndrome: Case Report

**DOI:** 10.4274/Jcrpe.970

**Published:** 2013-05-30

**Authors:** Emine Kartal Baykan, Mehmet Erdoğan, Samim Özen, Şükran Darcan, L. Füsun Saygılı

**Affiliations:** 1 Ege University Faculty of Medicine, İnternal Medicine Department of Endocrinology and Metabolism Unit, İzmir, Turkey; 2 Ege University Faculty of Medicine, Department of Pediatric Endocrinology and Metabolism Unit, İzmir, Turkey

**Keywords:** Romatase deficiency, delayed epiphyseal closure, estrogen deficiency, replacement therapy

## Abstract

Aromatase deficiency (AD) is a rare autosomal recessive inheritance syndrome. Its worldwide incidence is unknown, and there are few case reports in the literature. Aromatase dysfunction develops due to CYP19A1 gene mutation and a decrease in estrogen synthesis. Estrogen deficiency can induce delayed epiphyseal closure, eunuchoid body habitus, osteopenia, and osteoporosis in both genders. Our patient was a 27-year-old male who presented with bone pain, recurrent bone fractures associated with minimal trauma starting in puberty, and a progressive increase in height. Laboratory tests revealed that the blood levels of follicle-stimulating hormone and luteinizing hormone were above normal, testosterone level was normal, and estrogen was undetectable. Plain bone radiography of the left wrist and hand demonstrated that the epiphyses were still unfused. Lumbar osteoporosis was detected in bone densitometry. In the genetic analysis, homozygous R375H guanine-adenine (G-A) mutation was detected in the CYP19A1 gene, and a diagnosis of AD was reached. Treatment with 25 µg transdermal estradiol was started. All family members were examined. Homozygous R375H G-A mutation was detected in the patient’s younger brother. Heterozygous R375H G-A mutation was found in his mother, father, and older brother. In conclusion, this AD patient requires lifetime estrogen replacement in order to provide sufficient bone mineralization, to reduce the risk of bone fractures, and to lead a healthy life. The best method to prevent the possible complications is to diagnose the AD syndrome at early ages and to provide adequate estrogen replacement starting at puberty.

**Conflict of interest:**None declared.

## INTRODUCTION

Aromatase deficiency (AD) is a rare syndrome. Its incidence is not known worldwide, and there are only a limited number of case reports in the literature. In these patients, due to CYP19A1 gene mutation, abnormal protein synthesis occurs leading to aromatase dysfunction and consequently to a decrease in estrogen synthesis (1). Due to estrogen deficiency, disorders of sex development and progressive virilization at puberty develop in females. In the males, prepubertal development is normal. Delayed epiphyseal closure, eunuchoid body habitus, osteopenia, and osteoporosis develop in both genders (2). In this presentation, in the context of the case report of a patient with aromatase deficiency-related delayed epiphyseal closure and osteoporosis, we discuss the approach to the clinical diagnosis as well as to the treatment of this syndrome.

## CASE REPORT

**Figure 1:**
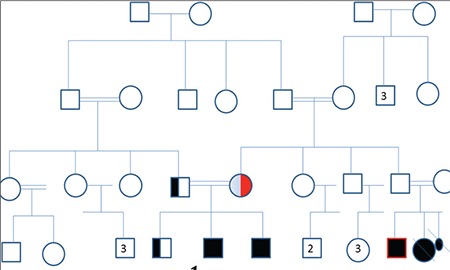
Pedigree of the patient

A 27-year-old male presented to our outpatient clinic with complaints of bone pain, recurrent forearm fracture associated with minimal trauma, and progressive increase in height. In his history, the pregnancy period of the patient’s mother was reported to be trouble-free, but since adequate information could not be obtained, it is not known whether virilization during pregnancy developed or not. The pedigree of the patient is shown in Figure 1. The patient was born by normal vaginal delivery with a normal birth weight in the 39th week of pregnancy. His development was reported to be normal during childhood and at puberty. First, a right forearm fracture developed after minimal trauma at age 15, and later, right and left forearm fractures occurred with minimal trauma at ages 19 and 24. A left forearm radiography performed at age 24 showed osteopenia, and the epiphyses were detected to be open. A diagnosis of osteoporosis was made based on bone densitometry performed in the medical facility to which he applied at age 26 with the complaint of pain in the bones, and alendronate 70 mg/week was started. After nearly one year of treatment, due to the progression of osteoporosis and progressive increase in height noted on follow-up, the patient was referred to our center for further evaluation. In his family history, the mother and father were first-degree cousins and he had two brothers. There was no infertility, primary amenorrhea, or hirsutism history in the family, but there was a history of kidney stones in two babies (relatives) who died in their early infancy (1-2 week). Our patient was born with ambiguous genitalia.

On physical examination, blood pressure was 125/75 mmHg, pulse rate 79/minute. His height was 187 cm (height of mother: 155 cm, height of father: 176 cm; target height: 172 cm). His weight was 90 kg, and body mass index was 25.7. Waist circumference was 102 cm, stroke distance 200 cm, ratio of upper part of body to lower body 0.85. A eunuchoid body habitus was noted. Secondary sexual characteristics were normal. The length and thickness of the penis was normal. Testicular volume, measured with an orchidometer, was >20 mL. No gynecomastia, goitre, acanthosis nigricans, or acromegalic appearance were noted.

Results of laboratory tests are presented in Table 1. As will be noted, follicle-stimulating hormone (FSH) and luteinizing hormone (LH) levels were above normal; testosterone level was normal, while estrogen was undetectable. Dehydroepiandrosterone sulphate, 17 hydroxyprogesterone, parathyroid hormone, thyroid-stimulating hormone and free thyroxine levels were all normal. Growth hormone basal level was 0.32 ng/mL. Following glucose loading for the oral glucose tolerance test, growth hormone level was 0.25 ng/mL. Insulin-like growth factor-1 level was 332 ng/mL (normal ranges for this age: 93-575 ng/mL). Total cholesterol and triglyceride concentrations were high; serum HDL concentration was low. Fasting blood glucose and oral glucose tolerance test yielded normal values. In semen analysis, sperm count was 56 million/mm with 40% motility. In liver ultrasonography, grade one hepatosteatosis was detected. Karyotype analysis showed a 46,XY pattern.

**Figure 2:**
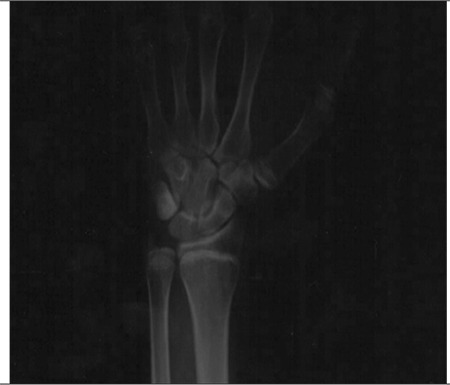
Plain bone radiography of the left wrist

Plain bone radiography of the left wrist and hand demonstrated open metacarpal and phalangeal epiphyses, and the bone age was estimated as 15 years (Figure 2). Bone densitometer revealed osteoporosis at the lumbar region (L1-L4 T score - 3.68, Z score - 3.68).

**Figure 3:**
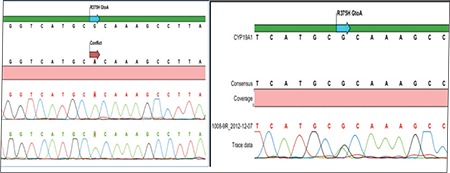
A) Analysis of genetic mutation: Homozygous R375HG-A mutation (CYP19A1gene); B) Analysis of genetic mutation:Heterozygous R375H G- A mutation (CYP19A1gene)

Genetic analysis was performed in the genetics laboratories of Ege University Faculty of Medicine. A homozygous R375H guanine-adenine (G-A) mutation was detected in the CYP19A1 gene (Figure 3a). The genetic analysis of the family members revealed a homozygous R375H G-A mutation in the younger brother and heterozygous R375H G-A mutation in the mother, father, and older brother (Figure 3b).

After the patient and his relatives were informed about the disease, 25 µg transdermal estradiol was started once in every three days. In the third month of the treatment, FSH, LH, and testosterone levels were found to be decreased. There was also a notable decrease in total cholesterol and triglyceride levels and an increase in HDL level. An increase in serum alkaline phosphatase and osteocalcin levels were also detected. 

## DISCUSSION

AD is a rare syndrome characterized by a decrease in estrogen synthesis due to aromatase dysfunction caused by CYP19A1 gene mutation. In 1995, AD and aromatase gene mutation were detected as an autosomal recessive disease in a male for the first time (3). Eight new male cases were reported between 1995 and 2010. The effect of estrogen on the reproductive system of women has been known for many years, however, the effects of estrogen in men and women outside of the reproductive system have only been recently identified (4). The effects of estrogen on the reproductive system in men have been clarified only within the past 10 years (5). At puberty, growth and bone mutation depend on androgen in both genders. Androgen turns into estrogen by aromatase in men. Estrogen levels increase due to increasing androgens at puberty and lead to bone maturation and ossification at the epiphyseal cartilages (6). In AD syndrome, estrogen deficiency causes delayed epiphyseal closure, eunuchoid body habitus, osteopenia, and osteoporosis that develop in both genders (2).

The mother who is expecting a child that has AD develops progressive virilization (increase in acne, voice thickening, cliteromegaly, frontal baldness, and facial hirsutism) during pregnancy. Due to placental aromatase deficiency, the transformation of androgen into estrogen decreases, and thus high levels of androgen exist in maternal blood. The virilization findings in the mother regress after the birth of the child (7). It is not known whether virilization had developed in the mother of the case presented in this report. In males, clinical findings of AD are not found in the newborn, and development is normal until puberty. Clinical signs of AD occur in puberty and especially in late puberty. Diagnosis is generally established after the second decade. Clinical and radiological findings include delay in bone maturation, lack of epiphyseal closure, continuation of linear growth, tall stature, eunuchoid body habitus, genu valgum, bone pain, osteopenia, and osteoporosis. Abnormal spermatogenesis, oligospermia, increase in testis volume, and cryptorchidism are often present. Metabolic syndrome findings such as abdominal obesity, insulin resistance, impaired glucose tolerance, acanthosis nigricans, non-alcoholic fatty liver, and dyslipidemia develop at early ages (1,4,8). The development of the patient we present was normal until puberty. His linear growth continued after puberty so that he got taller than the target height of his family. He had a eunuchoid body habitus.

Final diagnosis of AD is established by carrying out CYP191 mutation analysis through genetic analysis (9). Karyotype analysis must also be performed in order to eliminate chromosomal anomalies (10). In our case, 46,XY, homozygous R375H G-A mutation was detected in the CYP19A1 gene.

Treatment consists of estrogen replacement. Bone maturation and mineralization are normalized through estrogen treatment. Fast bone maturation is noted in 6-9 months when estrogen is used in high doses (25-50 µg/day) (11,12). 25 µg transdermal estradiol treatment was given every three days in our patient. In AD, estrogen treatment also normalizes gonadotropin secretion, glucose metabolism and liver functions, decreases lipids and insulin levels (13,14,15). In the third month of the treatment, decreases in FSH, LH, testosterone, total cholesterol and triglyceride levels, and increase in HDL level were detected in our patient. The increase in serum alkaline phosphatase and osteocalcin levels were linked to increase in remodeling activity within the bone. During the treatment, gynecomastia, hyperprolactinemia, or sexual dysfunction did not develop. No change was detected in testicular volume or semen analysis. The patient continues to be followed-up with 25 µg transdermal estradiol treatment.

In conclusion, in AD patients, estrogen replacement should be continued lifetime in order to provide sufficient bone mineralization, to reduce the risk of bone fracture, and for a healthy life. The best method to prevent complications is to diagnose the AD syndrome at early ages and to initiate adequate replacement as early as puberty.

## Figures and Tables

**Table 1:**
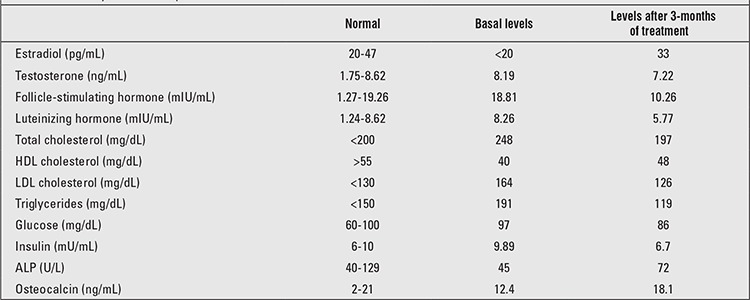
Laboratory results of the patient
