# Aromatase Deficiency, a Rare Syndrome: Case Report

**Published:** 2013-09-18

**Authors:** Elisa Pignatti, Manuela Simoni, Vincenzo Rochira

**Affiliations:** 1 Department of Biomedical, Metabolic and Neural Sciences, University of Modena & Reggio Emilia, Modena Italy; 2 Center for Genomic Research, University of Modena and Reggio Emilia; 3 Integrated Department of Medicine, Endocrinology and Metabolism, Geriatrics, Azienda USL of Modena, Modena, Italy and repeated later at Ege University.

## ERRATUM

The manuscript entitled “Aromatase Deficiency, a Rare Syndrome: Case Report” by authors “Emine Kartal Baykan, Mehmet Erdoğan, Samim Özen, Şükran Darcan, L.Füsun Saygılı” in JCRPE in Jun 2013 has reported that the genetic analysis of the patient was done at Ege University in Turkey. After the publication of the manuscript the editorial board of the Journal was informed that the genetic analysis was initially done by

Elisa Pignatti, MD, PhD^1,2^; Manuela Simoni, MD, PhD^1,2,3^; Vincenzo Rochira, MD, PhD^1,3^

^1^Department of Biomedical, Metabolic and Neural Sciences, University of Modena & Reggio Emilia, Modena Italy;

^2^Center for Genomic Research, University of Modena and Reggio Emilia,

^3^Integrated Department of Medicine, Endocrinology and Metabolism, Geriatrics, Azienda USL of Modena, Modena, Italy andrepeated later at Ege University.

Editorial Board

JCRPE

